# Influences of TiO_2_ and SiO_2_ nanofillers on the mechanical performance of a maxillofacial silicone elastomer after two years of outdoor tropical weathering: An in vitro study

**DOI:** 10.1371/journal.pone.0357673

**Published:** 2026-09-03

**Authors:** Belal Elmarhoumy, Mahmoud Salloum, Thamir Bahattab, Hussein Alhelaly, Mohammed Sghaireen, Matheel AL-Rawas, Johari Yap Abdullah, A. S. M. Rafiul Haque, Mohammed Mousa

**Affiliations:** 1 School of Dental Sciences, Universiti Sains Malaysia, Kota Bharu, Kelantan, Malaysia; 2 Department of Prosthodontics, Faculty of Dentistry, Sinai University, Kantara Branch, Ismailia, Egypt; 3 Department of Clinical Dental Sciences, Faculty of Dentistry, Zarqa University, Zarqa, Jordan; 4 Jeddah Specialist Dental Center, Jeddah Second Health Cluster, Ministry of Health, Saudi Arabia; 5 Mahayel Specialist Dental Center, Aseer Health Cluster, Ministry of Health, Saudi Arabia; 6 Department of Prosthetic Dental Sciences, College of Dentistry, Jouf University, Sakakah, Al Jowf, Saudi Arabia; 7 Dental Research Unit, Center for Transdisciplinary Research (CFTR), Saveetha Dental College, Saveetha Institute of Medical and Technical Sciences, Saveetha University, Chennai, India; 8 Department of Dental Anatomy, Udayan Dental College, Boalia, Rajshahi, Bangladesh; 9 Department of Restorative Dentistry, College of Dentistry, Ajman University, Ajman, United Arab Emirates; National Chung Cheng University College of Engineering, TAIWAN

## Abstract

The present study evaluated the influence of 3% and 10% (by weight) silicon dioxide (SiO2) and titanium dioxide (TiO2) nanoparticles (NPs) on the tear strength, tensile strength, and elongation at break of a room-temperature vulcanizing (RTV) maxillofacial silicone elastomer (MFSE; A-2000) after two years of natural tropical weathering. A total of 160 specimens (80 dumbbell-shaped and 80 trouser-shaped) were allocated to non-weathered and weathered groups, each divided into five subgroups (n = 8): unfilled control, 3% SiO2, 10% SiO2, 3% TiO2, and 10% TiO2. Weathered specimens were exposed outdoors in a hot, humid tropical climate for two years. Mechanical properties were measured using a universal testing machine. Data were analyzed using two-way ANOVA followed by Tukey HSD post-hoc tests (α = 0.05). In non-weathered specimens, 3% SiO2 increased tear strength by 34% (17.89 ± 0.31 vs. 13.38 ± 1.07 N/mm; *P* < 0.001) and tensile strength by 35% (4.28 ± 0.27 vs. 3.16 ± 0.13 MPa; *P* < 0.001) compared with the unfilled control. After natural tropical weathering, only the 3% SiO2 group maintained a significantly higher tear strength (14.03 ± 2.10 N/mm) than the unfilled group (11.09 ± 1.53 N/mm; *P* = 0.007). Weathering significantly reduced tear strength in all NP-filled groups (*P* < 0.001) but increased tensile strength across all groups (P < 0.01), with the smallest increase observed in the 10% TiO2 group (+10.1%; *P* = 0.004). Elongation at break decreased significantly in all groups following weathering (*P* < 0.001); however, NP-filled groups retained substantially higher elongation values (losses of 13.1–17.6%) than the unfilled control (loss of 51.1%). In conclusion, the incorporation of 3% SiO2 NPs significantly enhanced the mechanical properties of A-2000 MFSE both before and after two years of natural tropical weathering, whereas higher loading (10%) offered no additional benefit. These findings support the use of low-content SiO2 NPs to improve the long-term durability of maxillofacial prostheses.

## Introduction

Maxillofacial prostheses are used to restore facial defects resulting from trauma, oncological resection, or congenital anomalies. The ideal prosthetic material must exhibit adequate mechanical properties and maintain dimensional and chemical stability under environmental exposure to ensure satisfactory function and prolonged clinical service [1,2]. Room-temperature vulcanizing (RTV) maxillofacial silicone elastomers (MFSEs), particularly A-2000, are widely used due to their favorable handling characteristics, biocompatibility, and acceptable mechanical performance [3–5]. Nevertheless, prolonged exposure to natural weather, especially in tropical climates characterized by high ultraviolet (UV) radiation, elevated humidity, and marked temperature fluctuations, can degrade silicone polymers, leading to reduced tear resistance, loss of flexibility, and compromised prosthesis integrity [6–9].

To mitigate such degradation, the reinforcement of MFSEs with inorganic fillers has been extensively investigated. Among the available options, silicon dioxide (SiO2) and titanium dioxide (TiO2) nanoparticles (NPs) have received particular attention because they enhance mechanical performance through effective interfacial bonding with the silicone matrix [10–12]. Previous studies have reported that adding low percentages (≤ 3 wt%) of these NPs enhances tensile strength, tear strength, and hardness under laboratory conditions or after artificial aging [13–15]. A variety of fillers, including TiO2, titanium silicate (TiSiO₄), zinc oxide (ZnO), zirconium orthosilicate (ZrSiO₄), SiO2, yttrium oxide (Y2O₃), barium sulfate (BaSO₄), thixotropic agents, and silver and zinc particles, have been incorporated into MFSEs to improve their properties [10–12,16,17]. These fillers may be introduced in either micro- or nano-form [16,17]. NPs have consistently provided superior mechanical properties compared with microparticles, including higher tensile and tear strengths, greater hardness, and improved elongation percentage [10,18]. Despite these advances, no filler system has yet met universally accepted clinical guidelines.

SiO2 can be used either in its pure form or combined with other metal oxides to improve resistance to heat, chemicals, thermal expansion, and abrasion [19,20]. Different forms of SiO2 NPs have been evaluated as fillers at various weight percentages (1–3 wt%) to improve the mechanical properties of MFSEs. Fumed SiO2 has been shown to enhance the surface hardness of RTV MFSEs (A-2000, A-2186, and A-2006) more effectively than silane-treated SiO2, TiO2, or unfilled controls after two years of dark storage [21,22]. Similarly, heat-vulcanized MFSEs (Cosmesil M-511) reinforced with SiO2 exhibited superior mechanical and surface properties compared with the corresponding unfilled material [23].

TiO2 is a white, odorless, opaque mineral that occurs in several crystalline forms, of which rutile and anatase are the most relevant [20]. Its principal function in polymer systems is to impart whiteness and opacity, particularly when used in powder form. TiO2 NPs have been added to MFSEs at various concentrations to enhance both optical and mechanical properties. The addition of TiO2 NPs can improve the optical performance of polymers through quantum effects arising from the strong interfacial interaction between the organic polymer and the NPs [11]. In addition to these optical benefits, TiO2 has been reported to improve the mechanical properties of both heat-vulcanized (Cosmesil) and room-temperature-vulcanized (VST50F) MFSEs when added at concentrations of 0.20–0.25 wt% [11,24].

To the best of our knowledge, previously published studies have primarily examined the addition of low percentages of TiO2 and SiO2 NPs (0.5%, 1.0%, 1.5%, 2.0%, 2.5%, or 3.0% by weight) to MFSEs [10,19,25]. Furthermore, most of these investigations evaluated the influence of NPs immediately after fabrication, after dark storage, or after artificial aging protocols [12,18,21,22]. To date, no study has assessed the impact of a high percentage of NPs on the mechanical properties of MFSEs over extended periods of natural tropical weathering. For maxillofacial prosthodontists to reliably predict the clinical performance of these prostheses, it is essential to understand how highly filled MFSEs behave under realistic environmental conditions. Therefore, the present study was designed to evaluate the mechanical properties of an RTV MFSE (A-2000) incorporating low (3%) and high (10%) concentrations of TiO2 and SiO2 NPs after two years of natural tropical weathering. It was hypothesized (H₀) that neither high NP content nor two years of natural tropical weathering would significantly affect the mechanical properties of the tested MFSE.

## Materials and methods

### Specimen preparation

**Fig 1:**
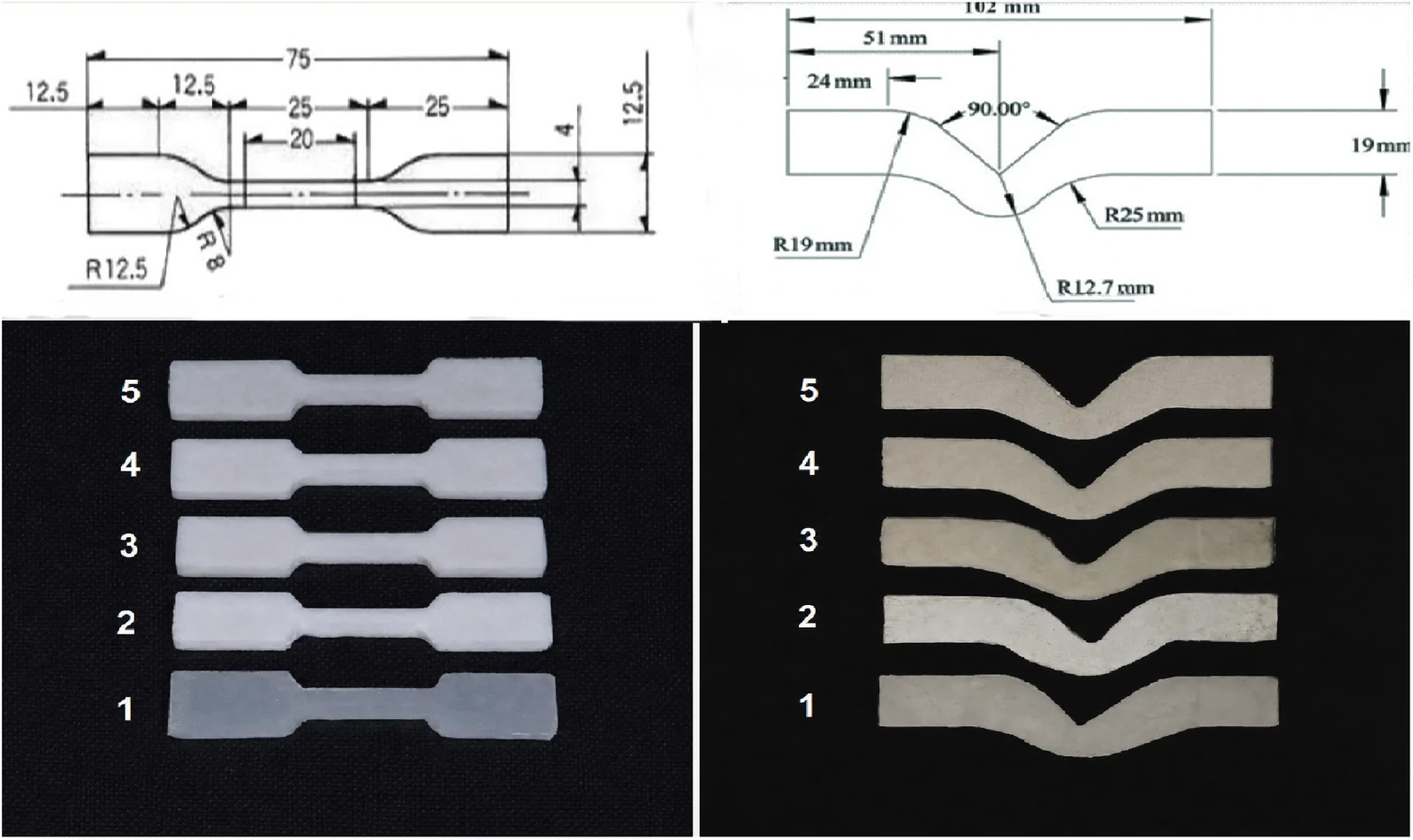
Representative specimens: dumbbell (left) and trouser (right) shapes. Labels indicate: 1, unfilled; 2, TiO2-filled (10%); 3, TiO2-filled (3%); 4, SiO2-filled (10%); 5, SiO2-filled (3%).

A total of 160 specimens were fabricated from RTV A-2000 platinum-catalyzed silicone (Factor II, Inc., Lakeside, AZ, USA), comprising 80 Type-II dumbbell specimens (in accordance with ASTM D412-16) and 80 Type-C trouser specimens (in accordance with ASTM D624) (Fig 1). Each specimen shape was randomly divided into two main groups—non-weathered and weathered (n = 40 each)—and each of these groups was further subdivided into five subgroups (n = 8) according to filler composition: unfilled (control), 3% SiO2, 10% SiO2, 3% TiO2, and 10% TiO2 NPs (American Elements, Los Angeles, CA, USA; particle size < 50 nm). The sample size was calculated using G*Power software (α = 0.05, power = 0.80, effect size f = 0.25, 10 groups, and 2 tests), yielding a required total of 80 specimens per mechanical test with an actual power of 85.8%.

NPs were weighed on an electronic digital balance (Sartorius BSA323S-CW; Göttingen, Germany) to achieve the specified weight percentage of the total silicone base. The NPs were then manually incorporated into the silicone base and mixed for 20 min to achieve visual homogeneity. The catalyst was subsequently added at a 1:1 ratio, and the mixture was vacuum-mixed using an electronic mixer (Hoover Mixyvac T; Manfredi, Pinerolo, Italy) for 30 min to ensure complete homogeneity and to eliminate air bubbles introduced during hand mixing. The mixed material was poured into stone molds using a low-speed electric oscillator (JG203; Zhermack S.p.A., Badia Polesine, Italy), transferred to a hydraulic press (660 psi), and allowed to polymerize for 72 h at room temperature. All specimens were visually inspected for defects; those exhibiting air bubbles or dimensional deviations ≥ 10% from the specifications were excluded and replaced.

### Weathering exposure

Weathered specimens were placed on glass exposure racks in the Health Campus garden of Universiti Sains Malaysia (Kelantan, Malaysia; latitude 6° N) from September 2021 to August 2023. Daily environmental parameters, including ambient temperature, relative humidity, UV index, rainfall, and wind speed, were recorded throughout the exposure period using the AccuWeather monitoring platform. Non-weathered (control) specimens were stored in a dehumidified cabinet at 23 ± 2 °C for the same period. Following weathering, specimens were gently cleaned with natural mineral water (Spritzer, Perak Darul Ridzuan, Malaysia) for 10 min and air-dried at room temperature.

### Mechanical testing

Tear strength was measured on trouser-shaped specimens using a universal testing machine (AG-20 KN; Shimadzu, Kyoto, Japan) at a crosshead speed of 50 mm/min. Tear strength was calculated as the force required to propagate the tear divided by the specimen thickness.

Tensile strength and elongation at break were determined on dumbbell-shaped specimens using the same universal testing machine at a crosshead speed of 5 mm/min, in accordance with ISO 37:2005. All specimen dimensions were measured with a digital vernier caliper (Mitutoyo Corp., Kawasaki, Japan) prior to testing. The operator performing the mechanical tests was blinded to group allocation.

### Statistical analysis

Data were analyzed using SPSS software (version 27.0; IBM Corp., Armonk, NY, USA). Normality of distribution was verified using the Shapiro–Wilk test. Two-way analysis of variance (ANOVA) was performed with weathering condition (non-weathered vs. weathered) and filler type (unfilled, 3% SiO2, 10% SiO2, 3% TiO2, 10% TiO2) as independent variables, separately for tear strength, tensile strength, and elongation at break. When significant interactions were detected, simple main effects were examined. Pairwise post-hoc comparisons were conducted using Tukey HSD tests (α = 0.05). Effect sizes were reported as partial eta squared (η²) for all significant effects.

## Results

### Environmental conditions

During the two-year outdoor exposure period, the mean ambient temperature was 24.7 °C (range 24.4–30.5 °C), the mean UV index was 9 (high), the mean relative humidity was 88%, the cumulative rainfall was 8,678 mm, and the mean wind speed was 9.77 km/h.

### Tear strength

Two-way ANOVA revealed significant main effects of filler type (*P* < 0.001; η² = 0.72) and weathering condition (*P* < 0.001; η² = 0.68), as well as a significant filler × weathering interaction (*P* = 0.004; η² = 0.12; Table 1). Among non-weathered specimens, the 3% SiO2 group exhibited the highest tear strength (17.89 ± 0.34 N/mm), which was significantly greater than that of the unfilled control (13.36 ± 1.16 N/mm; *P* < 0.001) and all other filler groups. The 10% SiO2 group (17.35 ± 1.07 N/mm) and the 3% TiO2 group (16.07 ± 1.29 N/mm) also demonstrated significantly higher tear strength than the unfilled control (*P* < 0.05), whereas the 10% TiO2 group (13.89 ± 1.43 N/mm) did not differ significantly from the unfilled control (*P* = 0.09) (Table 2).

**Table 1:** Two‑way ANOVA summary for tear strength.

Source	SS	df	MS	F	*P*	Partial η²
Weather	339.9	1	339.9	207.2	<0.001	0.75
Filler	145.2	4	36.3	22.1	<0.001	0.56
Weather×filler	50.73	4	12.7	7.7	<0.001	0.31
Error	114.8	70	1.64			

SS: Sum of Squares; df: Degrees of freedom; MS: Mean square. Weathering: F(1, 70)=207.2, *P* < 0.001. Filler: F(4, 70)=22.1, *P* < 0.001. Interaction (Weathering × Filler): F(4, 70)=7.7, *P* < 0.001.

**Table 2:** Means, post-hoc groupings, and the effect of natural tropical weathering on tear strength (N/mm).

Filler	NonWeathered	Weathered	Differences	Δ (loss)	t(70)	*P* (Bonferroni)
Nonfilled	13.38 ± 1.07^c^	11.09 ± 1.53^b^	−2.29	−17.1%	−3.58	0.002
3% SiO_2_	17.89 ± 0.31^a^	14.03 ± 2.10^a^	−3.86	−21.6%	−6.03	<0.001
10% SiO_2_	17.35 ± 1.07^ab^	11.26 ± 1.27^b^	−6.09	−35.1%	−9.51	<0.001
3% TiO_2_	16.07 ± 1.29^b^	10.24 ± 1.18^b^	−5.83	−36.3%	−9.1	<0.001
10% TiO_2_	13.89 ± 1.43^c^	11.35 ± 0.74^b^	−2.54	−18.3%	−3.97	0.003

Different superscript letters (a, b, c) within the same column indicate statistically significant differences between filler formulations within that specific weathering condition (P < 0.05, Tukey HSD post-hoc test); (a) denotes the highest and (c) the lowest value.

**Fig 2:**
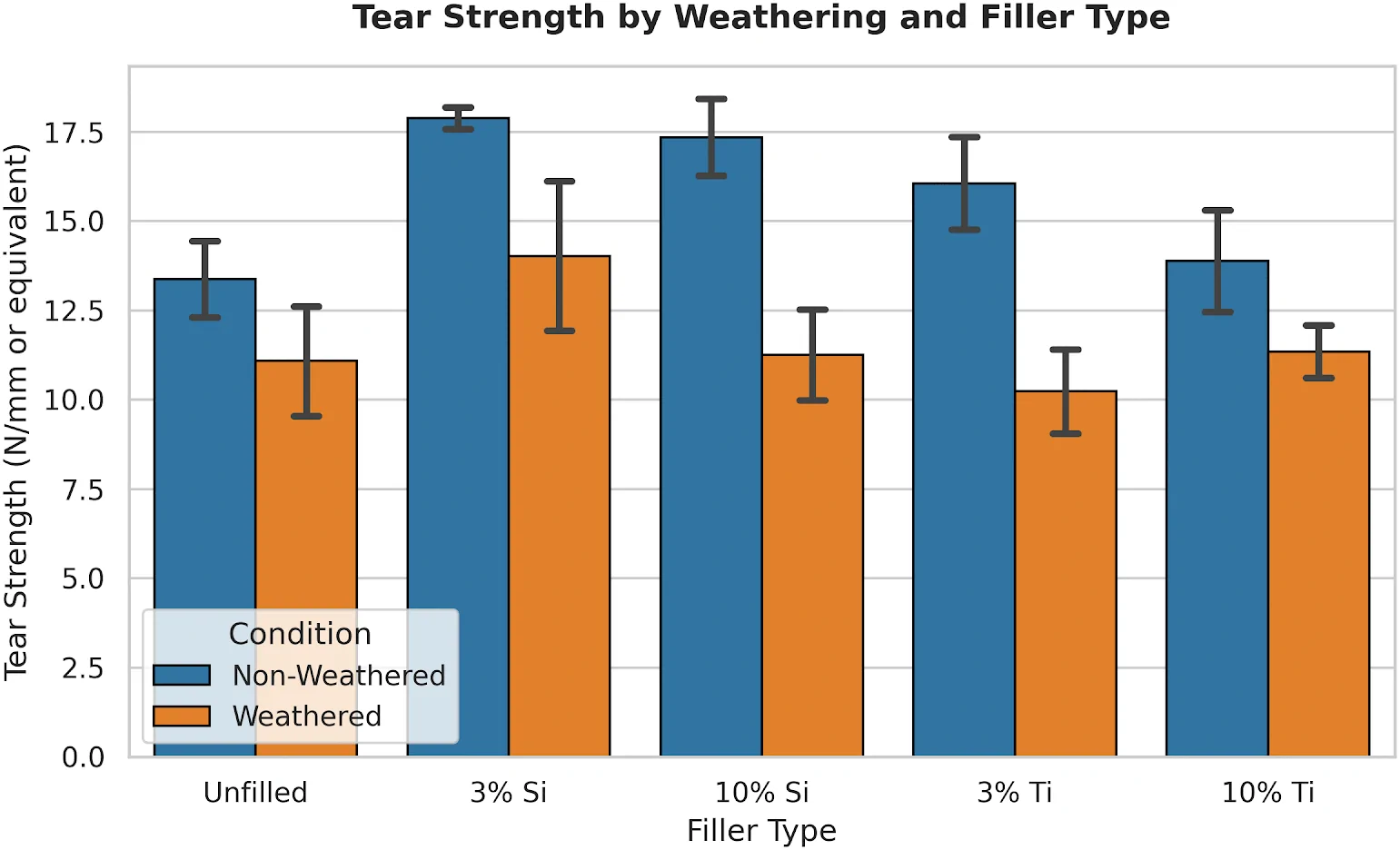
Effect of natural tropical weathering on the tear strength of A-2000 MFSE with various NP concentrations.

After two years of natural tropical weathering, only the 3% SiO2 group retained a significant advantage over the unfilled control (14.03 ± 2.10 vs. 11.09 ± 1.53 N/mm; *P* = 0.002). All other filled groups exhibited tear strength values comparable to those of the weathered unfilled control (*P* > 0.05). Weathering significantly reduced tear strength in all filled groups (*P* < 0.05), with percentage losses ranging from 21.6% (3% SiO2) to 36.3% (3% TiO2). The unfilled control showed a non-significant reduction of 17.1% (*P* = 0.09; Fig 2).

### Tensile strength

Two-way ANOVA demonstrated significant main effects of filler type (*P* < 0.001; η² = 0.64) and weathering condition (*P* < 0.001; η² = 0.63), together with a significant interaction (*P* = 0.002; η² = 0.27; Table 3). In non-weathered specimens, the 3% SiO2 group exhibited the highest tensile strength (4.28 ± 0.27 MPa), significantly greater than that of all other groups (*P* < 0.001). The 10% TiO2 (3.74 ± 0.31 MPa) and 3% TiO2 (3.63 ± 0.18 MPa) groups also displayed significantly higher tensile strength than the unfilled control (3.16 ± 0.13 MPa; *P* < 0.05), whereas the 10% SiO2 group (3.39 ± 0.16 MPa) did not differ significantly from the unfilled control (*P* = 0.07; Table 4).

**Table 3:** Two-Way ANOVA Summary for Ultimate Tensile Strength.

Source	SS	df	MS	F	P	Partial η²
Weather	7.77	1	7.76	118.09	<0.001	0.63
Filler	8.13	4	2.03	30.95	<0.001	0.64
Weather×filler	1.69	4	0.42	6.43	<0.001	0.27
Error	4.6	70	0.066			

SS: Sum of Squares; df: Degrees of freedom; MS: Mean square. Weathering: F(1, 70) =118.1, *P* < 0.001. Filler: F(4, 70)=30.95, *P* < 0.001. Interaction (Weathering × Filler): F(4, 70)=6.43, *P* < 0.001.

**Table 4:** Means, post-hoc groupings, and the effect of natural tropical weathering on ultimate tensile strength (MPa).

Fillers	NonWeathered	Weathered	Differences	Δ (loss)	t(70)	*P* (Bonferroni)
Nonfilled	3.16 ± 0.13^d^	4.35 ± 0.29^b^	+1.19	+37.7%	9.3	<0.001
3% SiO_2_	4.28 ± 0.27^a^	4.83 ± 0.34^a^	+0.55	+12.8%	4.28	<0.001
10% SiO_2_	3.39 ± 0.16 cd	3.85 ± 0.14^c^	+0.46	+13.6%	3.61	<0.001
3% TiO_2_	3.63 ± 0.18^bc^	4.17 ± 0.3^bc^	+0.53	+14.7%	4.16	<0.001
10% TiO_2_	3.74 ± 0.31^b^	4.12 ± 0.33^bc^	+0.38	+10.1%	2.96	0.004

Groups sharing the same superscript letter within a column are not significantly different (*P* ≥ 0.05); (a) denotes the highest and (d) the lowest value. All groups showed a significant increase in tensile strength after natural tropical weathering.

**Fig 3:**
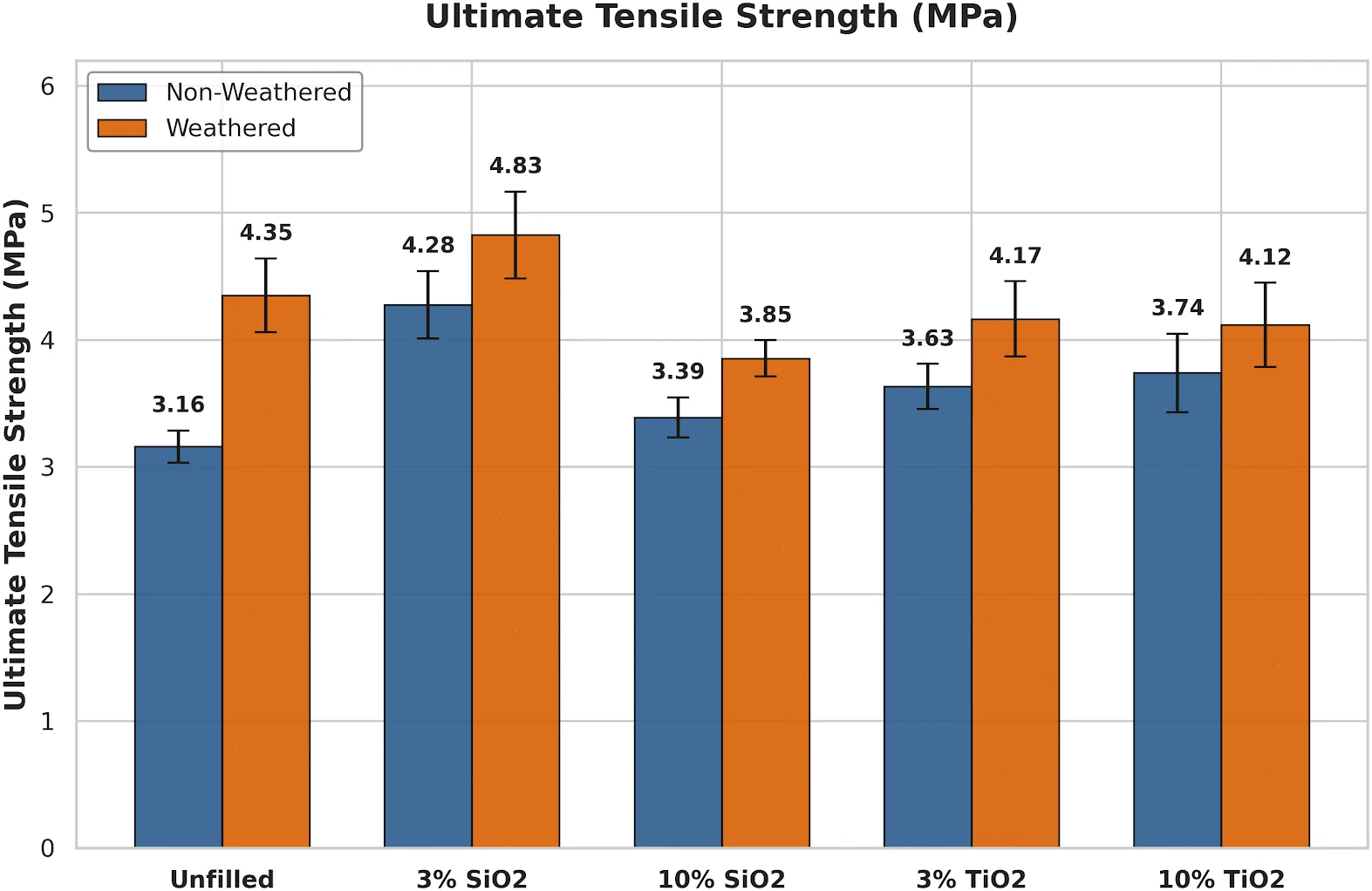
Effect of natural tropical weathering on the tensile strength of A-2000 MFSE with various NP concentrations.

Natural tropical weathering resulted in a significant increase in tensile strength across all groups. The unfilled control showed a 37.7% increase, from 3.16 ± 0.13 to 4.35 ± 0.29 MPa (*P* < 0.001). The 3% SiO2 group remained the highest after weathering (4.83 ± 0.34 MPa), being significantly greater than all other groups (*P* < 0.001). The 10% TiO2 group exhibited the smallest percentage change (+10.1%; 3.74 ± 0.31 vs. 4.12 ± 0.33 MPa; *P* = 0.004; Fig 3).

### Elongation at break

Two-way ANOVA revealed significant main effects of filler type (*P* < 0.001; η² = 0.57) and weathering (*P* < 0.001; η² = 0.68), together with a significant interaction (*P* < 0.001; η² = 0.43; Table 5). In non-weathered specimens, no significant differences in elongation at break were observed among the groups (*P* = 0.072), although the 3% SiO2 group displayed the highest numerical value (253.9 ± 20.4) and the unfilled control the lowest (231.3 ± 16.6; Table 6).

**Table 5:** Two-way ANOVA summary for elongation at break (%).

Source	SS	df	MS	F	P	Partial η²
Weather	59287.3	1	59287.34	149.49	<0.001	0.68
Filler	36686.4	4	9171.59	23.13	<0.001	0.57
Weather×filler	20572.7	4	5143.18	12.97	<0.001	0.43
Error	27761	70	396.59			

SS: Sum of Squares; df: Degrees of freedom; MS: Mean square. Homogeneity of variances was confirmed by Levene’s test. Weathering: F(1, 70)=149.5, *P* < 0.001. Filler: F(4, 70)=23.1, *P* < 0.001. Interaction (Weathering × Filler): F(4, 70)=13, *P* < 0.001.

**Table 6:** Simple main effects: filler comparisons within each weathering condition (Tukey HSD, α = 0.05) and weathering comparisons within each filler (Bonferroni correction for 5 tests) for elongation at break (%).

Fillers	NonWeathered	Weathered	Differences	Δ (loss)	t(70)	*P* (Bonferroni)
Nonfilled	231.3 ± 16.6^a^	113.1 ± 3.5^c^	−118.18	−51.1%	−11.87	<0.001
3% SiO_2_	253.9 ± 20.4^a^	212.6 ± 16.6^a^	−41.30	−16.3%	−4.15	<0.001
10% SiO_2_	232.6 ± 46.6^a^	191.7 ± 13.8^b^	−40.88	−17.6%	−4.11	<0.001
3% TiO_2_	240 ± 15.6^a^	208.5 ± 3.9^ab^	−31.51	−13.1%	−3.16	0.012
10% TiO_2_	242.7 ± 12.6^a^	202.3 ± 14.3^ab^	−40.36	−16.6%	−4.05	<0.001

Groups sharing the same superscript letter within a column are not significantly different (*P* ≥ 0.05); (a) denotes the highest and (c) the lowest value. All groups showed a significant decrease in elongation at break after natural tropical weathering.

**Fig 4:**
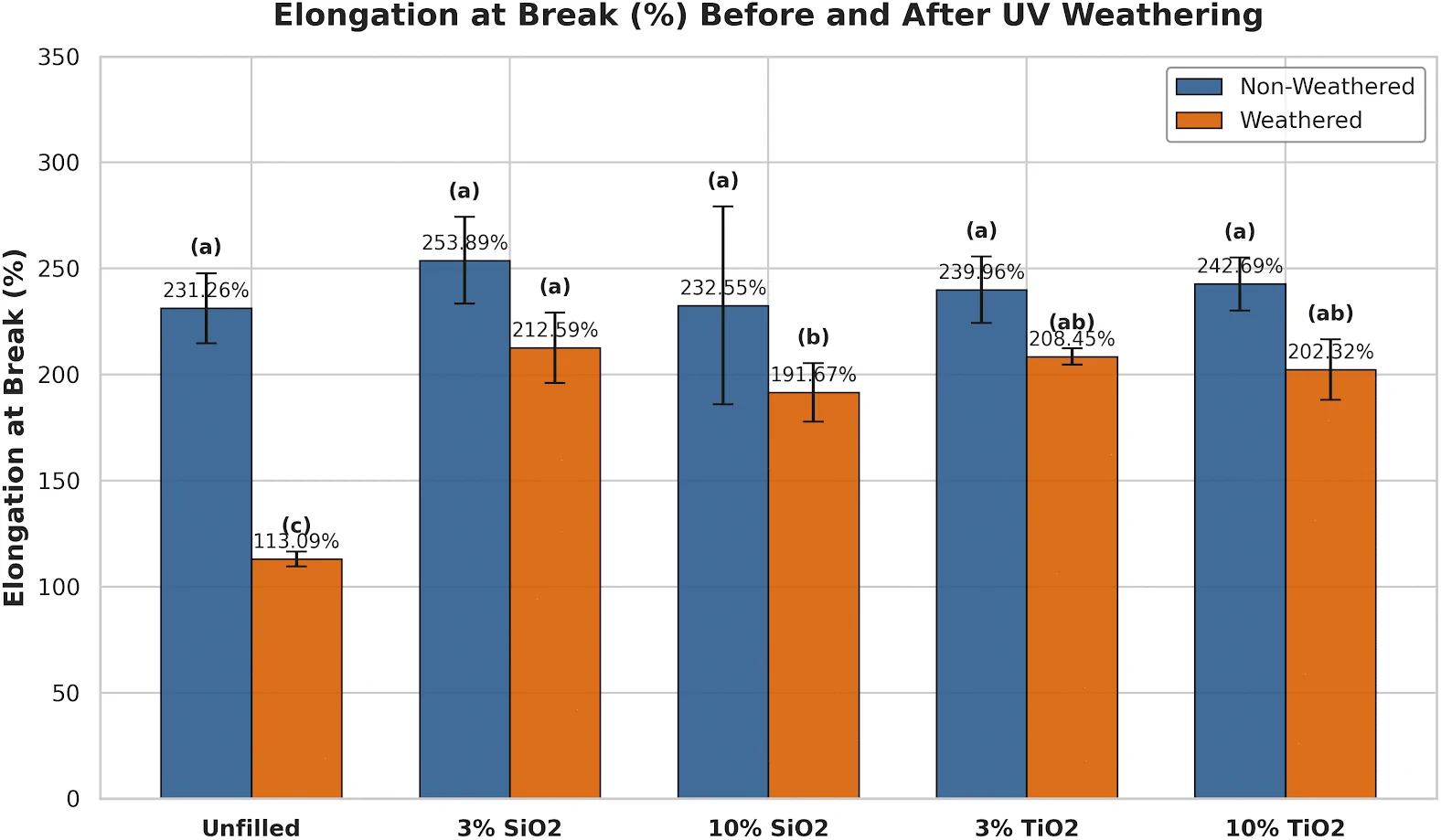
Effect of natural tropical weathering on the elongation at break of A-2000 MFSE with various NP concentrations.

After two years of natural tropical weathering, elongation at break decreased significantly in all groups (*P* < 0.001). The unfilled control exhibited the greatest reduction (from 231.3 ± 16.6% to 113.1 ± 3.5%; a loss of 51.1%). All filled groups retained significantly higher elongation values than the weathered unfilled control (*P* < 0.001), with the 3% SiO2 group showing the highest post-weathering elongation (212.6 ± 16.6%). Percentage losses in the filled groups ranged from 13.1% (3% TiO2) to 17.6% (10% SiO2), which were substantially smaller than the loss observed in the unfilled control (Fig 4).

## Discussion

The present study evaluated the long-term (two-year) effect of low (3%) and high (10%) concentrations of SiO2 and TiO2 NPs on the mechanical properties of an RTV MFSE (A-2000) exposed to a natural tropical environment. The results demonstrated that both filler type and concentration significantly influenced the mechanical properties, and that two years of natural tropical weathering altered these properties in a filler-dependent manner. Accordingly, the null hypothesis was rejected.

The superior performance of the 3% SiO2 group under both non-weathered and weathered conditions can be attributed to optimal NP dispersion and strong interfacial bonding with the silicone matrix [22]. At low concentrations, NPs are homogeneously distributed, creating a large interfacial area that restricts polymer chain mobility, thereby enhancing tear and tensile strength. The high specific surface area and abundant silanol groups on SiO2 facilitate hydrogen bonding with the silicone backbone, further improving reinforcement [20]. Conversely, at higher NP concentrations, agglomeration is more likely to occur, generating stress-concentration points and reducing reinforcement efficiency [25]. Although TiO2 NPs also exerted a reinforcing effect at 3%, they appeared less effective than SiO2, most likely due to weaker interfacial adhesion and a greater tendency to agglomerate within the nonpolar silicone matrix [21]. These findings are consistent with previous reports that documented improved mechanical properties of MFSEs reinforced with up to 3 wt% NPs under laboratory or artificial aging conditions [13–15].

The observed decrease in tear strength and elongation at break after natural tropical weathering is consistent with the well-established degradation mechanisms of silicones under UV radiation, moisture, and thermal cycling. Photodegradation generates free radicals that can cleave polymer chains, thereby reducing molecular weight and crosslink density [26]. The tropical climate of the exposure site, characterized by a high mean UV index (9) and elevated relative humidity (88%), likely accelerated these processes. The fact that the 3% SiO2 group partially preserved its tear strength, remaining significantly higher than that of the unfilled control after weathering, suggests that well-dispersed NPs can function as UV absorbers or free-radical scavengers, thereby mitigating chain scission [27].

An unexpected finding was the significant increase in tensile strength observed across most groups after weathering, particularly in the unfilled control (+37.7%). This phenomenon may be attributed to post-curing of the silicone under outdoor conditions. Prolonged exposure to heat and UV radiation can promote additional crosslinking reactions among residual reactive groups, increasing tensile strength at the expense of elongation [28]. This interpretation is supported by the concurrent marked decrease in elongation within the same groups. Notably, the 10% TiO2 group was the only formulation that did not exhibit a significant change in tensile strength following weathering. This exception likely reflects excessive filler agglomeration at higher loadings, which may hinder uniform crosslinking and block UV penetration, thereby attenuating both post-curing and photodegradation. Most previously published studies used NP concentrations ≤ 3 wt% [10,19,25], and our results confirm that higher filler contents (10%) offer no benefit and may even interfere with the beneficial weathering-induced crosslinking.

It is important to contextualize the present findings within the broader debate regarding artificial aging versus natural weathering. Most previously published investigations of nano-reinforced MFSEs have relied on artificial aging protocols, typically involving accelerated exposure in weather-o-meters, UV-A/UV-B chambers with condensation cycles (e.g., ASTM G154), xenon-arc devices (e.g., ASTM G155/ISO 4892−2), or thermocycling at controlled temperatures for periods ranging from a few hundred to a few thousand hours [12,14,17,18]. These protocols offer the advantages of reproducibility, time efficiency, and the ability to isolate specific degradation factors such as UV wavelength, temperature, or moisture. Nevertheless, they represent an oversimplified simulation of the actual environment because they cannot faithfully reproduce the synergistic and cyclical interactions among UV radiation, thermal cycling, precipitation, atmospheric pollutants, biological contaminants, and diurnal humidity variations that occur in natural service conditions [29].

In contrast, natural tropical weathering, as applied in the present study, exposes specimens to the full spectrum of environmental stressors encountered by clinical prostheses in equatorial regions, including sustained high UV indices (mean = 9), consistently high humidity (mean = 88%), heavy cumulative rainfall (8,678 mm over two years), and wide diurnal temperature fluctuations. Such conditions typically induce more complex degradation pathways than accelerated laboratory protocols, including hydrolytic scission of siloxane bonds, oxidative chain cleavage, and simultaneous post-curing of residual reactive groups. This may explain the apparently paradoxical simultaneous increase in tensile strength and decrease in tear strength and elongation observed in the present study, an outcome that is not commonly reported in short-term artificial aging studies, which usually document uniform reductions in all mechanical parameters [17,18]. Consequently, while artificial aging remains a valuable screening tool, natural weathering studies provide more clinically relevant data on the long-term durability of MFSEs. Future work should ideally compare identical formulations under both aging modalities to establish reliable correlation factors between accelerated and real-time exposure.

The substantial preservation of elongation in the NP-filled groups after weathering is particularly noteworthy. Whereas the unfilled control lost 44.9% of its elongation capacity, the filled groups lost only 13.1–17.6% of theirs. This indicates that NP reinforcement effectively preserves the flexibility and extensibility of the silicone elastomer even after prolonged environmental exposure. The 3% TiO2 group showed the best preservation of elongation (loss of 13.1%), suggesting that TiO2 NPs may provide superior protection against embrittlement, possibly by shielding the polymer matrix from UV-induced photodegradation.

For maxillofacial prostheses, tear strength is a critical property in thin marginal regions such as the eyelids and nasal alae, which are repeatedly removed and reinserted [29]. Our results indicate that adding 3% SiO2 can maintain tear strength above that of the unfilled control even after two years of outdoor service, potentially extending the prosthesis’s useful lifespan. Elongation at break, which reflects flexibility and ease of placement, was also better preserved in the filled groups. Although natural tropical weathering reduced elongation across all groups, the NP-filled materials remained superior to the unfilled control, suggesting that even a 10% NP loading provides some protection against embrittlement. Clinically, the unexpected weathering-induced increase in tensile strength may appear beneficial; however, it is accompanied by reductions in tear strength and elongation, both of which are more relevant to the long-term durability of thin prosthetic margins. Therefore, overall mechanical durability is best optimized by the addition of 3% SiO2, which balances preservation of tear strength and elongation while still permitting some degree of beneficial post-curing.

The present study evaluated only one silicone formulation (A-2000) and two NP types; therefore, the findings may not be generalizable to other MFSEs or filler systems. Chemical and morphological characterization (e.g., FTIR and SEM) of the same specimens has been reported in a previously published companion study to confirm the degradation mechanisms and filler dispersion patterns [5]. Although the natural weathering exposure applied here reflects realistic service conditions, it inevitably introduces uncontrolled environmental variables; parallel artificial aging tests would be helpful in isolating specific degradation factors. Additionally, clinical factors such as perspiration, sebum, cosmetic products, and mechanical abrasion were not simulated. Further in vivo investigations are needed to correlate the observed mechanical changes with actual clinical performance.

## Conclusion

Within the limitations of this study, the incorporation of 3% SiO2 NPs significantly improved the tear and tensile strengths of A-2000 RTV MFSE, both before and after two years of natural tropical weathering, when compared with the unfilled material; the 3% TiO2 group ranked next. Increasing NP content to 10% provided no additional benefit over the 3% concentration for either filler type. Two years of natural tropical weathering reduced tear strength and elongation at break but increased tensile strength in most groups, with the 3% SiO2 formulation displaying the most favorable overall mechanical profile. These findings support the use of low-content (3%) SiO2 NPs as a simple and effective strategy to enhance the long-term mechanical durability of maxillofacial silicone prostheses used in challenging tropical climates.

## Supporting information

S1 Data Belal et al Raw data. (XLSX)
